# A novel supergene regulates alternative dispersal morphs without variation in social organization in the ant *Myrmecina nipponica*

**DOI:** 10.1098/rsbl.2026.0475

**Published:** 2026-10-28

**Authors:** Ermae Liprado, Isaac Planas-Sitjà, Yu Okamura, Adam L. Cronin

**Affiliations:** ^1^ Department of Biological Sciences, Tokyo Metropolitan University, Hachioji, Japan; ^2^ Center for Interdisciplinary Theoretical and Mathematical Sciences (iTHEMS), RIKEN Wako Campus, 2-1 Hirosawa, Wako, Saitama 351-0198, Japan

**Keywords:** formicidae, polymorphism, spatially variable selection, dispersal

## Abstract

Polymorphisms in complex traits with discrete phenotypes are often underpinned by non-recombining complexes of co-adaptive genes, known as supergenes. In disparate lineages of ants, it is increasingly evident that supergenes are important in regulating intra-specific variation in social organization (number of reproductive queens in a colony). Variation in social organization in ants is often associated with variation in dispersal, with multiple-queen (polygynous) colony types exhibiting more limited dispersal than single-queen (monogynous) colony types. It has been suggested that this association may give rise to antagonistic selection, which in turn may help explain the multiple independent evolutions of social supergenes in ants. In this study, we provide evidence of an exception to this pattern in the form of a novel supergene in the ant *Myrmecina nipponica*. This species exhibits a dispersal polymorphism with winged and wingless queens but without variation in social structure, as all colonies have a single, reproductive queen. We show that the dispersal polymorphism in this species is underpinned by an approximately 6.8 Mb supergene novel to ants. Interestingly, this supergene is structurally and functionally different from that recently reported in another member of this genus, illustrating how patterns of supergene evolution can show marked intrageneric variation.

## 1. Introduction

Polymorphisms in complex traits with discrete phenotypes are often regulated by supergenes: non-recombining assemblages of co-adaptive loci that define alternative phenotypes. Supergenes underlie diverse traits in a broad range of taxa, from alternative modes of butterfly mimicry [1–3] to floral types in buckwheat [4] and migratory behaviours in cod [5]. Supergenes may be particularly important in highly social organisms because the interplay between individual and group-level phenotypes means that changes in one phenotype can lead to selection on others [6]. In the fire ant *Solenopsis invicta*, for example, a supergene influences traits including queen number and dispersal behaviour, worker and male body size and male sperm count [7–9]. These interactive effects at the group and individual levels may help explain a remarkable example of evolutionary convergence in ants, in which supergenes associated with social structure (whether colonies are headed by single or multiple queens) are thought to have evolved independently at least eight times [6,8,10–17].

Variation in social structure in ants is often associated with variation in dispersal. In a pattern known as polygyny syndrome, queens from polygynous (multi-queen) colonies are typically smaller and disperse locally relative to monogynous (single-queen) forms [18–20]. These traits interact through the effect of dispersal on kin structure, which can potentially lead to genetic integration through the selection of favourable trait combinations [21]. Thus, it is perhaps not surprising that supergene systems incorporating these traits have evolved independently numerous times in ants [6,10,16]. This has led some researchers to propose that antagonistic selection may be a common mechanism working to favour particular combinations of these traits [14,22–24]. Interestingly, current evidence indicates that either trait may pave the way for evolutionary integration of the other. For example, the social supergene evolved prior to the evolution of queen miniaturization in *Formica cinerea* [22], while the genetic architecture underpinning the dispersal polymorphism predates the evolution of the social supergene in *Myrmecina graminicola* [14]. Further comparative studies are thus needed to elucidate the importance of the interaction between social structure and dispersal in driving supergene evolution and how this might vary between species.

Ants of the genus *Myrmecina* exhibit variation in queen morphology associated with dispersal (winged and wingless queens) and colonies can contain one or more queens [14,25]. A recent study of *M. graminicola* by Mona *et al.* [14] revealed a complex supergene system in which an approximately 20 Mb social supergene on chromosome 1 underpins the variation in social structure and queen morphology associated with dispersal (winged and wingless queens). The authors showed that the social supergene is of recent origin (approx. 1 Ma) relative to the polymorphism in queen morphology (approx. 20 Ma). The Asian sister species, *Myrmecina nipponica*, also exhibits variation in queen morphology and queen number, although it is reported to be functionally monogynous (single reproductive queen) rather than truly polygynous (multiple reproductive queens), as in *M. graminicola* [25,26]. These two sister species are thought to have diverged approximately 4–8 Ma [27], predating the evolution of the social supergene but after the evolution of the genetic architecture underpinning the dispersal polymorphism in *M. graminicola*. This provides an opportunity to investigate the outcomes of supergene evolution among closely related species and whether there is any evidence of intrageneric variation in the patterns of supergene loss or convergent evolution, as has recently been reported among *Formica* ants [28]. In this study, we used genome-wide SNPs to investigate the genetic basis of trait polymorphism in *M. nipponica* and assess how the differences in life-history traits between this species and *M. graminicola* are reflected in the supergene system.

## 2. Methods

### (a) Sampling and molecular data acquisition

We collected entire colonies of *M. nipponica* from five localities near Chitose in Hokkaido, Japan, between 2018 and 2022 (supplementary material, figure S1). In this area, colonies of two queen types co-occur: ergatoid queens (worker-like, permanently wingless and with local dispersal) and alate queens (queens with enlarged thoraces, wings that are abscised once colonies are established and the potential for long-range dispersal); however, queens of both types are typically not found in the same colony. These queens are easily distinguished even when their wings are abscised (see [29]). This species has been reported to be functionally monogynous [25], although polygyny occurs in other members of the genus [14]. We made no *a priori* assumptions and considered that all multi-female colonies were potentially polygynous. Thus, we classified the colonies by queen number (single queen (SQ) or multi-queen (MQ)) and queen type (alate queen (AQ) or ergatoid queen (EQ)).

We used restriction site-associated DNA sequencing (RAD-seq) to obtain genome-wide single nucleotide polymorphism (SNP) data from 55 queens (11 AQ (9 SQ and 2 MQ) and 44 EQ (25 SQ and 19 MQ) from 50 colonies (supplementary material, table S1, data file 1) [30]. SNP calling was performed in *ipyrad* v.0.9.81 [31] based on the reference genome of the sister species *M. graminicola* [14]. Because of the trade-off between SNP quality and quantity and our need for both properties, we generated two datasets. For analyses focused on delimiting genomic regions, we generated a ‘broad’ dataset that focused on SNP number to maximize mapping resolution by filtering the output of *ipyrad* with VCFtools [32] to remove indels, set a minimum minor allele frequency of 0.05, remove low-quality individuals (>80% missing data) and remove sites with >25% missing data, yielding a dataset of 12 017 SNPs (supplementary material, data file 2) [30]. For all other analyses, we generated an additional ‘strict’ dataset that focused on SNP quality to limit biases introduced by low-quality SNPs by further filtering the 12 017 SNP dataset with a minimum sequencing depth of 20, generating a dataset of 4398 loci (supplementary material, data file S3; also see table S2 for characteristics of each dataset) [30].

A single genetic population was inferred based on the 12 017 SNP dataset using the *find.clusters* function of the R package *adegenet* [33], and inter-site *F_ST_* estimated with the R package *hierfstat* [34] was low (< 0.032; supplementary material, table S3); thus, individuals from all five collection localities were pooled for downstream analyses.

### (b) Inference of social structure

To confirm the social structure of *M. nipponica* in our study population, we dissected 127 queens from 53 colonies (including 39 colonies that were not used for genetic analysis), scoring individuals for ovarian development, insemination status and evidence of previous ovarian activity (presence of yellow bodies). We then classified individuals as ‘reproductively active’ if they fulfilled the condition ((inseminated + developed ovaries) *or* evidence of previous laying), where ‘developed ovaries’ were classified as those rated as 4 or 5 on a scale of 0–5 (also see supplementary material, supplementary methods and figure S2).

### (c) Genomic basis of trait variation

We followed Mona *et al.* [14] in defining a candidate supergene region as one hosting SNPs that, with respect to the polymorphic character expressed: (i) show *F_ST_* above the 95th percentile of the empirical distribution, (ii) have strong linkage disequilibrium (LD) relative to the genome-wide average, and (iii) can clearly distinguish groups on the first axis of a PCA. We investigated the association between molecular data and queen type (AQ/EQ) and queen number (SQ/MQ) by (i) calculating pairwise *F_ST_* among individuals in these groups for each SNP in VCFtools (broad dataset), (ii) quantifying LD among SNPs mapped to the *M. graminicola* genome assembly using R package *SNPRelate* [35] (broad dataset), and (iii) performing PCAs of SNPs using the R package *pcadapt* [36] (strict dataset). Using this approach, we identified a region of interest (ROI) associated with queen morphology on chromosome 4 (see §3). To help infer genotypes in the region of interest, we quantified heterozygosity by SNP for this region in VCFtools for SNPs showing the highest association with queen morphs from the broad dataset (*n* = 41 SNPs with *F_ST_* >0.75 for the comparison of queen morphs). We inferred the gene content of the ROI by conducting BLASTx searches of annotated genes located in the ROI of the reference genome against the NCBI nr database. We then performed gene ontology (GO) annotation using Blast2GO [37] and assessed GO enrichment in the ROI by comparing the frequency of GO terms for genes in the ROI with that of the rest of the genome using FDR-corrected Fisher's exact tests. To explore potential synteny between the ROI and supergenes associated with alternative dispersal morphs in other ants (supplementary material, table S4), we aligned the sequences with MUMmer4 [38] using default parameters and a minimum span of 200bp and visualized the results with a circos plot generated with the R *circlize* package [39].

## 3. Results

A comparison of genetic genome-wide SNPs between ergatoid and alate queen morphs revealed a large genomic region exhibiting high differentiation on chromosome 4 (figure 1a,c). This ‘region of interest’ (ROI), spanned approximately 6.8 Mb from 12 163 509 to 18 983 110 bp, as defined by the locations of the first and last SNP of the cluster in chromosome 4 showing *F_ST_* above the 95th quantile. By contrast, there was no evidence for genetic differentiation associated with variation in queen number (figure 1b). This latter finding is perhaps unsurprising, given the purported functional monogyny of this species. Functional monogyny was confirmed for our study population through dissections, which revealed comparable ovarian development in reproductively active individuals in single-queen and multi-queen colonies and a single reproductively active female in multi-queen colonies (*n* = 26 EQ and 1 AQ; supplementary material, figure S3, data file 4) [30].

**Figure 1.**
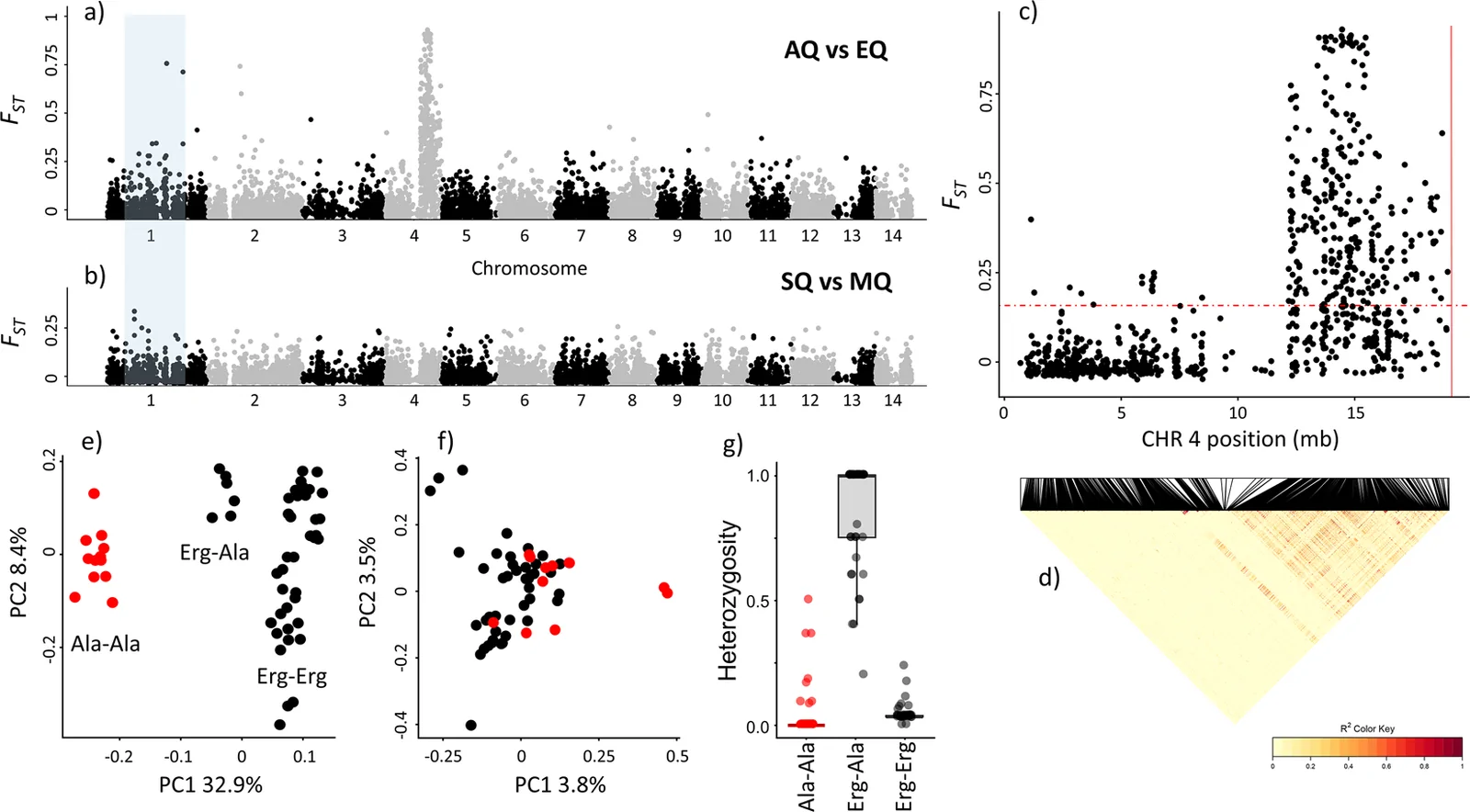
Plots of *F_ST_* for queen morphology (a) and queen number (b) by chromosome for SNPs aligned to the 14 chromosomes of the *M. graminicola* genome, with the social supergene region of *M. graminicola* on chromosome 1, highlighted in blue. (c) *F_ST_* of chromosome 4 SNPs; the red dotted line indicates the 95th quantile of the *F_ST_* distribution and the vertical red line indicates the position of the Awing gene in *M. gramincola*. (d) Plot of LD for chromosome 4 SNPs. (e) PCA coloured by queen type (red = AQs, black = EQs) and labelled by inferred genotype for SNPs in the ROI. (f) Corresponding PCA for whole-genome SNPs, excluding the ROI. (g) Boxplot of heterozygosity by SNP for individuals in each genotype cluster in figure 1e for SNPs with *F_ST_* >0.75 in figure 1c (*n* = 41; red = AQs, grey/black = EQs). Figures a–d and g are based on ‘broad’ 12 017 SNP dataset; figures e and f are based on ‘strict’ 4398 SNP dataset.

SNPs within the ROI exhibited a high degree of linkage disequilibrium relative to the rest of the chromosome (figure 1d; median LD of chromosome 4 among SNPs inside ROI = 0.068, outside = 0.012). We extended this analysis across all 14 chromosomes of the *M. graminicola* genome and found additional clusters of high LD SNPs on chromosomes 3 and 14 (supplementary material, figure S4). SNPs within clusters on chromosomes 4 and 3 (though not 14) showed evidence of linkage with scattered SNPs on other chromosomes, though not with each other (supplementary material, figure S5). PCAs of the high LD regions on chromosomes 3 and 14 showed strong clustering patterns, but with no relationship with the queen morph, queen number or site (supplementary material, figure S6).

EQ and AQ individuals were clearly distinguished in a PCA of ROI SNPs, whereas there was no differentiation among morphs for SNPs from the rest of the genome (figure 1e,f; also see supplementary material, figure S7 for PC1 versus PC3). The three clusters in figure 1e were consistent with alate queens being homozygous for the ROI and ergatoid queens heterozygous or homozygous for the alternate allele (figure 1g). We refer to these alleles as *Ala* and *Erg,* respectively, following Buschinger [40], who inferred a gene-regulated basis for queen polymorphism in *M. graminicola* using cross-breeding experiments. Heterozygous ergatoid individuals were thus notably rare and most were found at one site (supplementary material, figure S8).

Unlike *M. graminicola*, we found very little evidence for an influence of chromosome 1 on variation in queen number or dispersal in *M. nipponica*; only two SNPs had high *F_ST_* (approx. 0.75) for queen morphology within the region of the *M. graminicola* social supergene (figure 1a), neither of which was within the region of the insertion in *M. graminicola*. Interestingly, the *Awing* gene on chromosome 4 in *M. graminicola* mapped just outside the ROI in *M. nipponica* (figure 1c). A copy of this gene is part of an approximately 116kb insertion associated with queen polymorphism in chromosome 1 in *M. graminicola* (also see supplementary material, table S5).

Our BLAST/GO analysis identified 320 genes in the ROI, 282 of which were assigned to at least one GO term (supplementary material, data file 5) [30]. Four genes in this region were assigned to the ‘Cytochrome P450 (CYP) gene family’ (GO:0 006 082), members of which are conserved across insects and have been implicated in hormone metabolism and developmental regulation [41,42]. GO enrichment analysis identified 10 GO terms that were enriched relative to the broader genome, particularly for functions related to ‘cellular anatomical structure’ and ‘membrane’ (supplementary material, figure S9).

The comparison of genomic regions with other species exhibiting supergene polymorphisms indicated regions of overlap of the ROI with *S. invicta*, *Pogonomyrmex californicus* and *Temnothorax rugatulus*, but in all cases on chromosomes with no known association with the social/dispersal polymorphism (figure 2). The only overlap with known supergenes was a partial association with the social chromosome of *Formica selysi*, which is more distantly related to *Myrmecina* than any of the other species.

**Figure 2.**
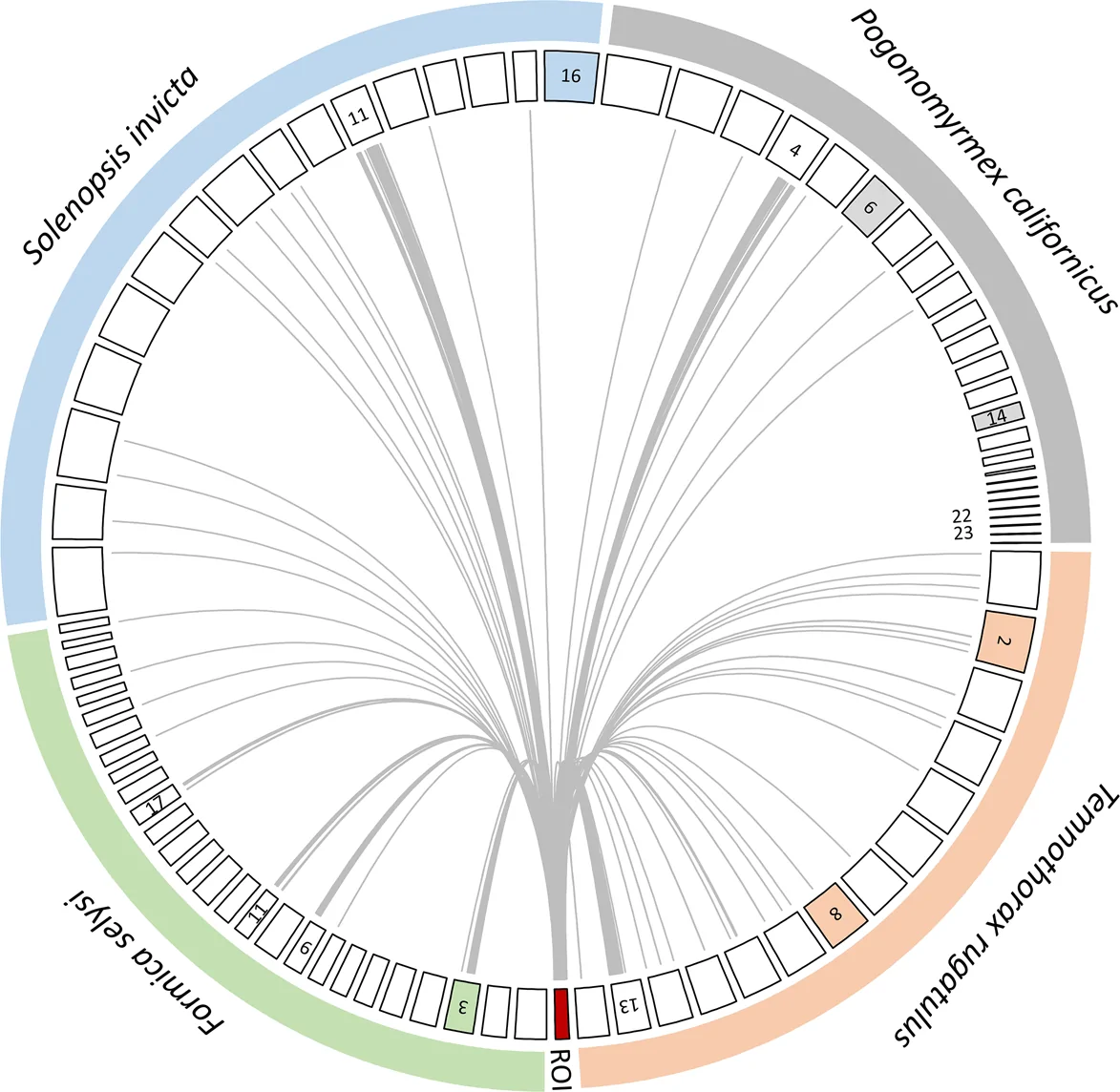
Circos plot of synteny between the ROI (based on the genome of *M. graminicola*) and genomes of other ant species exhibiting supergene-driven polymorphisms with variation in dispersal. For clarity, only chromosomes indicating synteny or known to be associated with supergene polymorphism are numbered (the latter highlighted in colour) and only the first 25 scaffolds of the genome for *P. californicus* are shown.

## 4. Discussion

We identified a large (approx. 6.8 Mb) region of chromosome 4 associated with a dispersal polymorphism (winged and wingless queen morphs) in *M. nipponica*. This region exhibits the characteristics of a supergene, making it the first example of a dispersal-related supergene polymorphism without variation in social organization in ants. Patterns of heterozygosity across the ROI suggest that alates are homozygous for the recessive supergene allele, whereas ergatoids are homozygous or heterozygous for the dominant allele.

This supergene appears to be novel in ants, as we found no broad synteny with known ant supergenes. Partial synteny was observed with the social chromosome of *Formica selysi* (CHR3) and CHR9, which is linked with queen miniaturization in *F. cinerea* [22]. However, these morphological polymorphisms are unlikely to share functional roots with *M. nipponica,* given the distant evolutionary relationship with *Formica* and the different forms of polymorphism (microgynes in *Formica* versus wingless, worker-like queens in *Myrmecina*). Notably, the supergene system in *M. nipponica* also appears to be functionally and structurally different from that recently reported for the sister species, *M. graminicola*, despite both species exhibiting similar winged and wingless queen morphs. Queen polymorphism in *M. nipponica* is linked to a supergene on chromosome 4, whereas in *M. graminicola,* it is associated primarily with the social supergene on chromosome 1. In *M. graminicola*, wing polymorphism is associated with an approximately 116 kb insertion in the social supergene, which was duplicated from chromosome 4 and encodes the *Awing* gene. Interestingly, the *Awing* gene on chromosome 4 in *M. graminicola* was mapped just outside the ROI in *M. nipponica*. However, as none of our SNPs mapped to the position of *Awing*, we cannot say whether this gene is present in *M. nipponica* or whether it is linked to the chromosome 4 supergene. We found no indication of a genetic association with variation in queen number in *M. nipponica*, which aligns with the functional monogyny that appears typical for this species. Antagonistic selection has been invoked as an explanation for the repeated evolution of supergenes integrating dispersal and social characteristics in ants [6,10,14,22,43]. However, in contrast to its sister species *M. graminicola*, the supergene-mediated dispersal polymorphism in *M. nipponica* was not followed by the evolution of a social supergene but appears to have remained stable in the absence of polygyny.

Various mechanisms for maintaining supergene polymorphism have been proposed in ants, including overdominance, negative frequency dependence and spatially variable selection (reviewed in [6,10]). In our study population, both supergene alleles were found at four of the five sites (supplementary material, figure S8) and gene flow between sites was largely unrestricted (supplementary material, table S3). However, only six of the 55 queens sampled were heterozygous for the supergene (compared to 11 Ala-Ala and 38 Erg-Erg individuals), considerably fewer than expected under panmixia and equal viability of different genotypes. This may argue against an effect of overdominance, but the veracity of this pattern should be confirmed over a broader scale, and the possibility of fitness differences between homozygous and heterozygous EQs should be investigated. A previous report of broad-scale geographic variation in queen morphs in *M. nipponica* [26] provides evidence of a possible influence of spatially variable selection, and we found evidence that genotypes were not evenly distributed at the local scale in our study population (supplementary material, figure S8). Spatially variable selection may also help maintain supergene-based social polymorphisms in other ants [44,45] and wing polymorphisms in other insects [46,47]. However, while environmentally-driven loss of flight is common in insects [48], the selective factors for social organisms, and particularly ants which fly only once during natal dispersal, may differ from those of other organisms [49]. For example, the loss of flight in ant queens may be a secondary consequence of selection for dispersing with (wingless) workers, as worker presence can aid overwintering survival and enhance early colony growth [50–52]. If spatially variable selection can help explain the maintenance of the supergene-based dispersal polymorphism in *M. nipponica*, it will be interesting to see how this compares mechanistically with *M. graminicola*, which also shows geographic variability in the frequency of queen morphs [27]. Assessing the role of other factors that can influence the frequency of supergene haplotypes, including recessive lethality, assortative mating and selfish genetic drive (e.g. [8,23,53–55]), requires further investigation, although it would seem that recessive lethality is unlikely, given that homozygotes of both genotypes were found (figure 1g; supplementary material, table S1).

A caveat of this study is our reliance on the genome of *M. graminicola* to map our SNPs. However, we anticipate that any discrepancies arising from this will be minimal, given the close affinity between these sister species (the two species are thought to have diverged recently (< 8 Ma) and *M. nipponica* was previously considered a subspecies of *M. graminicola*). Nonetheless, the patterns reported should be confirmed with higher-resolution genetic data and the construction of a genome for the focal species. Broader-scale sampling will also help divulge how supergene haplotypes vary with the geographic gradient in the frequency of different queen morphs in *M. nipponica* [26].

This study adds to the recent evidence from *Formica* ants indicating that supergene evolution can follow alternative trajectories in closely related species. Recent studies [16,28,56] have reported evidence of convergent evolution and differential loss of supergenes among closely related *Formica* ants. Here, we found that the genetic structure underpinning trait polymorphism also differs among sister species of *Myrmecina*. In *M. graminicola,* a duplication event led to an insertion that underpins dispersal polymorphism. This insertion was dated to approximately 20 Ma [14], predating the divergence of these species and indicating that the genetic basis for the dispersal polymorphism was present in the common ancestor. Divergence following speciation is thus a possible explanation for the structural and functional differences between these species. Under this scenario, the inserted region in *M. graminicola* became associated with the social structure supergene on chromosome 1, whereas in *M. nipponica*, the original copy of this region on chromosome 4 became elaborated with non-social traits. At present, however, we cannot confirm the presence of *Awing* in *M. nipponica* or rule out the possibility that the supergene reported here is the result of a novel and independent evolutionary event. Future analyses incorporating higher resolution data should be able to resolve this question and help elucidate the role of the high LD regions on other chromosomes in this species.

## Supplementary Material



## Data Availability

Data and supporting information for this article can be found on Figshare [30]. Supplementary material is available on Figshare [57].
