# Supplementary material for: A novel supergene regulates alternative dispersal morphs without variation in social organization in the ant Myrmecina nipponica
Source: Biol Lett. 2026 Oct 28;22(10):20260475. doi: 10.1098/rsbl.2026.0475 (PMC13725522; doi:10.1098/rsbl.2026.0475)
Supplement: Supplementary file 1 [file rsbl.2026.0475.zip › rsbl.2026.0475/rsbl20260475_si_001.docx]

**A novel supergene regulates alternative dispersal morphs without variation in social organisation in the ant *Myrmecina nipponica***

Ermae Liprado^1^, Isaac Planas-Sitjà^2^, Yu Okamura^1^, Adam L Cronin^1*^

**Supplementary Methods**

*Collection sites*

Ants were collected from five sites in Hokkaido, Japan, spread over a roughly linear transect of ~5km (figure S1). For further details see supplementary material, data file 1.

**Figure S1.** Map of collection sites. *(a)* Northern Japan, *(b)* Hokkaido showing area of Chitose (red square), *(c)* view of sites in Chitose area.

**Table S1**. Details of colonies used in genetic analysis. ID = individual ID; QT = queen type (AQ = alate queen, EQ = ergatoid (wingless) queen); CT = colony type; SQ = single queen, MQ = multiple queen; Site = collection site; Cluster = cluster in PCA in figure 1e, genotype = inferred supergene genotype based on PCA and analysis of heterozygosity.

| # | Year | ID | QT | CT | Site | Cluster | genotype |
| --- | --- | --- | --- | --- | --- | --- | --- |
| 1 | 2017 | 2017-23 | EQ | MQ | 3 | C | ERG/ERG |
| 2 | 2017 | 2017-24 | AQ | SQ | 3 | A | ALA/ALA |
| 3 | 2018 | cts2018-11 | EQ | SQ | 3 | C | ERG/ERG |
| 4 | 2018 | cts2018-12 | AQ | SQ | 3 | A | ALA/ALA |
| 5 | 2018 | cts2018-13 | EQ | SQ | 3 | C | ERG/ERG |
| 6 | 2018 | cts2018-15 | EQ | MQ | 3 | C | ERG/ERG |
| 7 | 2018 | cts2018-15 | EQ | MQ | 3 | C | ERG/ERG |
| 8 | 2018 | cts2018-16 | EQ | SQ | 3 | C | ERG/ERG |
| 9 | 2018 | cts2018-18 | EQ | SQ | 3 | C | ERG/ERG |
| 10 | 2018 | cts2018-3.1 | EQ | SQ | 2 | C | ERG/ERG |
| 11 | 2018 | cts2018-3.2 | EQ | SQ | 2 | C | ERG/ERG |
| 12 | 2018 | cts2018-3.3 | EQ | SQ | 2 | C | ERG/ERG |
| 13 | 2018 | cts2018-4.1 | EQ | SQ | 4 | B | ALA/ERG |
| 14 | 2018 | cts2018-4.2 | EQ | SQ | 4 | C | ERG/ERG |
| 15 | 2018 | cts2018-4.3 | EQ | SQ | 4 | B | ALA/ERG |
| 16 | 2018 | cts2018-8 | EQ | MQ | 3 | C | ERG/ERG |
| 17 | 2018 | cts2018-8 | EQ | MQ | 3 | C | ERG/ERG |
| 18 | 2000 | CTS2005 | EQ | SQ | 1 | C | ERG/ERG |
| 19 | 2000 | CTS2006 | EQ | MQ | 1 | C | ERG/ERG |
| 20 | 2000 | CTS2006 | EQ | MQ | 1 | C | ERG/ERG |
| 21 | 2000 | CTS2008 | AQ | SQ | 1 | A | ALA/ALA |
| 22 | 2000 | CTS2010 | EQ | SQ | 1 | C | ERG/ERG |
| 23 | 2000 | CTS2011 | EQ | SQ | 1 | C | ERG/ERG |
| 24 | 2000 | CTS2016 | EQ | SQ | 1 | C | ERG/ERG |
| 25 | 2000 | CTS2019 | EQ | SQ | 1 | C | ERG/ERG |
| 26 | 2000 | CTS2023 | EQ | SQ | 1 | C | ERG/ERG |
| 27 | 2000 | CTS2025 | EQ | MQ | 1 | C | ERG/ERG |
| 28 | 2000 | CTS2028 | EQ | MQ | 1 | C | ERG/ERG |
| 29 | 2000 | CTS2030 | EQ | MQ | 2 | C | ERG/ERG |
| 30 | 2000 | CTS2031 | EQ | MQ | 2 | C | ERG/ERG |
| 31 | 2000 | CTS2032 | EQ | SQ | 2 | C | ERG/ERG |
| 32 | 2000 | CTS2033 | EQ | MQ | 2 | C | ERG/ERG |
| 33 | 2000 | CTS2034 | EQ | MQ | 2 | C | ERG/ERG |
| 34 | 2000 | CTS2035 | EQ | MQ | 2 | C | ERG/ERG |
| 35 | 2000 | CTS2036 | EQ | SQ | 2 | C | ERG/ERG |
| 36 | 2000 | CTS2050 | EQ | MQ | 4 | C | ERG/ERG |
| 37 | 2000 | CTS2051 | EQ | SQ | 4 | B | ALA/ERG |
| 38 | 2000 | CTS2053 | EQ | MQ | 4 | B | ALA/ERG |
| 39 | 2000 | CTS2054 | EQ | MQ | 4 | B | ALA/ERG |
| 40 | 2000 | CTS2054 | EQ | MQ | 4 | C | ERG/ERG |
| 41 | 2000 | CTS2055 | EQ | SQ | 4 | C | ERG/ERG |
| 42 | 2000 | CTS2064 | AQ | SQ | 4 | A | ALA/ALA |
| 43 | 2000 | CTS2065 | EQ | SQ | 4 | C | ERG/ERG |
| 44 | 2022 | 2022-21 | EQ | SQ | 5 | C | ERG/ERG |
| 45 | 2022 | 2022-23 | EQ | SQ | 5 | C | ERG/ERG |
| 46 | 2022 | 2022-31 | EQ | MQ | 5 | C | ERG/ERG |
| 47 | 2022 | 2022-32 | EQ | SQ | 5 | C | ERG/ERG |
| 48 | 2022 | 2022-34 | AQ | SQ | 5 | A | ALA/ALA |
| 49 | 2022 | 2022-40 | AQ | MQ | 5 | A | ALA/ALA |
| 50 | 2022 | 2022-40 | AQ | MQ | 5 | A | ALA/ALA |
| 51 | 2022 | 2022-41 | AQ | SQ | 5 | A | ALA/ALA |
| 52 | 2022 | 2022-42 | AQ | SQ | 5 | A | ALA/ALA |
| 53 | 2022 | 2022-46 | AQ | SQ | 5 | A | ALA/ALA |
| 54 | 2022 | 2022-47 | AQ | SQ | 5 | A | ALA/ALA |
| 55 | 2022 | 2022-53 | EQ | SQ | 5 | B | ALA/ERG |

*Molecular data acquisition*

Heads and gasters were removed from ants and the remainder used for DNA extraction with Qiagen DNeasy Blood and Tissue Kits following the manufacturer’s instructions. DNA was digested with restriction enzyme Msel in a total reaction volume of 20 µL, then barcoded by individual and purified. Fragments were size-selected to between 300 and 450 bp with Pippin Prep (Sage Science, USA) and enriched through 14 PCR cycles. Samples were standardised to 50ng DNA/sample then RAD-seq library construction was undertaken using Multiplexed Shotgun Genotyping [1]. Paired-end 150 bp sequencing was performed with NovaSeq 6000 (Illumina, USA). Sequencing was performed by Adaptor Genomic Sciences LTD, Taiwan.

**Table S2**. Summary of molecular datasets. The broad dataset was filtered to remove indels, low quality individuals (>80%) missing data, and loci with >25% missing data, and set a minor allele frequency of 0.05. The strict dataset was additionally filtered by a minimum sequencing depth of 20. WG = whole genome SNPs, ROI = region of interest SNPs, (Ala-Ala, Ala-Erg, Erg-Erg) = ROI SNPs for individuals in the clusters in figure 1e. SNPs = number of SNPs in region, Depth = mean depth per loci, Missingness = mean proportion of missing data per loci.

| Dataset | Region | SNPs | Depth | Missingness |
| --- | --- | --- | --- | --- |
| Broad | WG | 12017 | 20.1 | 0.15 |
|  | ROI | 561 | 21.1 | 0.16 |
| Strict | WG | 4398 | 27.1 | 0.11 |
|  | ROI | 190 | 31.0 | 0.11 |
|  | Ala-Ala | 190 | 39.6 | 0.04 |
|  | Ala-Erg | 190 | 30.2 | 0.10 |
|  | Erg-Erg | 190 | 28.3 | 0.13 |

*Social structure*

While multiple queens can be found in colonies of *Myrmecina nipponica*, social structure in ants is determined by the number of reproducing queens in a colony, not the number of queens present. Thus, polygynous colonies are those which have more than one reproductive queen present, while monogyny, or functional monogyny, refers to those with a single queen or single reproductively active queen, respectively. Previous studies [2] have suggested that alate colonies of *M. nipponica* are monogynous, while ergatoid colonies may be monogynous or functionally monogynous. This characterisation of the species was based on analysis of colonies from one site (Tomakomai, near, but not contiguous with our Chitose site). To confirm that monogyny is the norm for our population, we dissected queens to determine reproductive status and investigate evidence for past reproductive activity.

*Dissection data and approach*

Queens were dissected under a Micronet microscope, scoring the following characteristics: i) insemination status (presence of sperm in the spermatheca), ii) indication of previous laying activity (presence of yellow bodies), iii) ovarian development (scored from undeveloped (0) to well developed (5); figure S2). In some cases, dissections were not possible for samples used in sequencing because individuals had been preserved in ethanol and were thus unsuitable for dissection. We therefore supplemented dissection data of sequenced individuals with additional fresh samples from the same population. In total, 127 queens were dissected from 53 colonies, comprising eight AQ colonies (7 SQ/1 MQ) and 45 EQ colonies (17 SQ/28 MQ); see electronic supplementary material, data file 4 for details.

Based on previous studies indicating that colonies are functionally monogynous in this species [2], we assumed colonies to be (functionally) monogynous unless there was clear evidence to the contrary. Thus, colonies were determined to be polygynous only if multiple, actively reproductive queens were present. We defined reproductively active queens as those with (sperm in the spermatheca AND well-developed ovaries) OR yellow-bodies indicative of previous egg-laying. We defined ‘well-developed’ ovaries as those with a score of 4 or 5 on our scale of 0-5.

**Figure S2.** Dissected ovaries of *M. nipponica* queens. A) with moderately developed ovaries (level 2), and also showing yellow-bodies (YB) and an inseminated spermatheca (SP). B) developed ovaries (level 4) and C) completely undeveloped ovaries (level 0).

*Genetic structure of sampled populations*

To assess the genetic structure of the five sampled population, we assessed genetic clustering with the *find.clusters* function of the R adegenet package using the 12017 SNP dataset, with a maximum of five clusters and retaining 40 PCs. This analysis indicated that individuals from all populations were part of a single genetic cluster. Analysis of inter-population Wier and Cockerham *F_st_* in the R *hierfstat* package based on SNPs on the whole genome SNPs indicated low differentiation among sites (Table S2).

**Table S3. Weir and Cockerham *F_st_* by collection site for 12017 whole genome SNPs**

| Site | 1 | 2 | 3 | 4 |
| --- | --- | --- | --- | --- |
| 2 | 0.014717 |  |  |  |
| 3 | 0.015813 | 0.012847 |  |  |
| 4 | 0.017541 | 0.016939 | 0.006810 |  |
| 5 | 0.031480 | 0.031940 | 0.017771 | 0.013564 |

**Table S4.** Details of ant genomes used in synteny analysis.

| **Species** | **GenBank** | **Citation** |
| --- | --- | --- |
| *Solenopsis invicta* | GCA_016802725.1 | [3] |
| *Formica selysi* | GCA_009859135.1 | [4] |
| *Pogonomyrmex californicus* | GCA_050084945.1 | [5] |
| *Temnothorax rugatulus* | GCA_057190385.1 | [6] |

**Supplementary Results**

*Social structure*

Of the 30 colonies containing multiple queens (1 AQ, 29 EQ), only one individual in each colony was classified as reproductive based on our criteria (supplementary material, data file 4). These findings indicate functional monogyny is predominant in the study population, and support an earlier study indicating *M. nipponica* is functionally monogynous [2]. This suggest queens in multi-queen colonies may be following delayed dispersal or inheritance strategies. In a few cases, individuals classified as reproductive because of the presence of yellow-bodies were scored as uninseminated during dissections. It seems unlikely that these individuals – as the sole reproductive present - had only been laying male-destined eggs, and thus this may indicate that insemination status was not always clear during dissections, and thus that insemination rates are underestimated, and/or that females may suffer from sperm depletion such that they appear uninseminated. In either case this does not alter the pattern of functional monogyny in these colonies. Dissections of queens from 53 colonies (1 AQ-MQ, 7 AQ-SQ, 29 EQ-MQ, 16 EQ-SQ) revealed similar patterns of ovarian development in single and multiple queen colonies (figure S2).

**Figure S3.** Distribution of ovary size classes for “reproductively active” (see above) females in multi-queen (hashed bars) and single-queen (solid colour) colonies for EQs (black) and AQs (red). Samples sizes are as follows: AQMQ 1, AQSQ 7, EQMQ 29, EQSQ 16.

**Figure S4**. Plots of LD for *M. nipponica* RAD SNPs mapped to all 14 chromosomes of the *M. graminicola* genome based on the ‘broad’ 12017 SNP dataset.

**Figure S5**. Linkage disequilibrium (LD) across chromosomes for SNPs mapped to the *M. graminicola* genome based on the broad (12017 SNP) dataset. Stronger colour indicates increasing LD from 0.5 to 1. Red bars in peripheral arcs indicate clusters of high LD SNPs within each chromosome (see figure S3).

**Figure S6**. PCAs of SNPS within the high LD regions in chromosomes 3 and 14 (see figure S3) indicating strong clustering patterns but with no clear association with queen morph or site. There was also no association with queen number (not shown).

**Figure S7**. PCA of ROI SNPs for the strict dataset showing PC1 vs PC3.

**Figure S8.** PCA of ROI SNPs coloured by collection site and labelled as in Figure 1e, indicating possible spatial structuring of different genomic clusters in the case of cluster B, left PC1 vs PC2 and right PC1 vs PC3.

**Table S5.** Genome mapping details for the ROI and notable gene regions in *M. graminicola* (Mona et al 2025).

| **Species** | **Location** | **CHR** | **Start** | **Finish** |
| --- | --- | --- | --- | --- |
| *M. nipponica* | ROI | 4 | 12,163,509 | 18,983,110 |
| *M. graminicola* | Social supergene | 1 | 5,940,000 | 26,615,000 |
|  | Duplicated region | 1 | 22,855,500 | 22,975,000 |
|  | AwingDPL (copy) | 1 | 22,962,299 | 22,969,129 |
|  | Duplicated region | 4 | 18,998,000 | 19,024,000 |
|  | Awing | 4 | 19,000,979 | 19,006,196 |

**Figure S9**. Results of GO enrichment analysis. The reference set was the entire genome without the ROI, while the test set was the ROI.

**References**

[1] Andolfatto, P., Davison, D., Erezyilmaz, D., Hu, T.T., Mast, J., Sunayama-Morita, T. & Stern, D.L. 2011 Multiplexed shotgun genotyping for rapid and efficient genetic mapping. *Genome Res.* **21**, 610-617.

[2] Murakami, T., Wang, L. & Higashi, S. 2000 Mating frequency, genetic structure, and sex ratio in the intermorphic female producing ant species *Myrmecina nipponica*. *Ecol. Entomol.* **25**, 341-347.

[3] Wang, J., Wurm, Y., Nipitwattanaphon, M., Riba-Grognuz, O., Huang, Y.-C., Shoemaker, D. & Keller, L. 2013 A Y-like social chromosome causes alternative colony organization in fire ants. *Nature* **493**, 664.

[4] Brelsford, A., Purcell, J., Avril, A., Van, P.T., Zhang, J., Brütsch, T., Sundström, L., Helanterä, H. & Chapuisat, M. 2020 An ancient and eroded social supergene is widespread across *Formica* ants. *Curr. Biol.* **30**, 304-311. e304.

[5] Errbii, M., Ernst, U.R., Lajmi, A., Privman, E., Gadau, J. & Schrader, L. 2024 Evolutionary genomics of socially polymorphic populations of *Pogonomyrmex californicus*. *BMC Biol.* **22**, 109.

[6] Darras, H., Yu, C., Kever, M., Stoldt, M., Feldmeyer, B. & Foitzik, S. 2026 Sequential origin of an alternative colony founding strategy driven by two supergenes in *Temnothorax* ants. *Mol. Biol. Evol.* **43**, msag115.
